# Adolescent and young adult preferences for financial incentives to support adherence to antiretroviral therapy in Kenya: a mixed methods study

**DOI:** 10.1002/jia2.25979

**Published:** 2022-09-15

**Authors:** Ingrid Eshun‐Wilson, Eliud Akama, Fridah Adhiambo, Zachary Kwena, Bertha Oketch, Sarah Obatsa, Sarah Iguna, Jayne L. Kulzer, James Nyanga, Everlyne Nyandieka, Ally Scheve, Elvin H. Geng, Elizabeth A. Bukusi, Lisa Abougi

**Affiliations:** ^1^ Division of Infectious Diseases, Department of Medicine Washington University in St. Louis St. Louis Missouri USA; ^2^ Research Care Training Program, Centre for Microbiology Research Kenya Medical Research Institute Kisumu Kenya; ^3^ Department of Obstetrics, Gynecology, and Reproductive Services University of California San Francisco California USA; ^4^ Division of Pediatrics University of Colorado Boulder Colorado USA

**Keywords:** preference, discrete choice, incentive, adolescent, HIV, antiretroviral

## Abstract

**Introduction:**

To develop a patient‐centred financial incentive delivery strategy to improve antiretroviral treatment adherence in adolescents and young adults (AYA) living with HIV in Kisumu, Kenya, we conducted a mixed methods study exploring preferences.

**Methods:**

A discrete choice experiment (DCE) and focus group discussion (FGD) were conducted simultaneously to identify preferences for five incentive delivery strategy features: value, eligibility, recipient, format and disbursement frequency. We used consecutive sampling to recruit AYA (14–24 years) living with HIV attending three health facilities in Kisumu, Kenya. We calculated mean preferences, willingness to trade, latent class membership and predictors of latent class membership. The FGD explored preferred incentive features, and, after deductive and inductive coding, qualitative findings were triangulated with DCE results.

**Results:**

Two hundred and seven AYA living with HIV (46% 14–17 years, 54% 18–24 years; 33% male sex, 89% viral load <50 copies/ml) were recruited to the study (28 October–16 November 2020). Two distinct preference phenotypes emerged from the DCE analysis (*N* = 199), 44.8% of the population fell into an “immediate reward” group, who wanted higher value cash or mobile money distributed at each clinic visit, and 55.2% fell into a “moderate spender” group, who were willing to accept lower value incentives in the form of cash or shopping vouchers, and accrued payments. The immediate reward group were willing to trade up to 200 Kenyan Shillings (KSH)—approximately 2 US dollars (USD)—of their 500 KSH (∼5 USD) incentive to get monthly as opposed to accrued yearly payments. The strongest predictor of latent class membership was age (RR 1.45; 95% CI: 1.08–1.95; *p* = 0.006). Qualitative data highlighted the unique needs of those attending boarding school and confirmed an overwhelming preference for cash incentives which appeared to provide the greatest versatility for use.

**Conclusions:**

Providing small financial incentives as cash was well‐aligned with AYA preferences in this setting. AYA should additionally be offered a choice of other incentive delivery features (such as mobile money, recipient and disbursement frequency) to optimally align with the specific needs of their age group and life stage.

## 1 INTRODUCTION

Although financial incentives have been extensively investigated to improve uptake of HIV prevention interventions and adherence to antiretroviral treatment (ART) in adults living with HIV, the use of this economic strategy to motivate adolescents and young adults (AYA) living with HIV to remain in HIV care and on treatment is less well understood. Through the provision of a financial reward to accomplish a specific task or behaviour, financial incentives aim to reinforce and shape healthier behaviour [1]. Individuals frequently weight present costs and benefits relative to those in the future and incentives can act as a “nudge” towards adopting a healthy behaviour by increasing its immediate benefits [2, 3, 4]. Financial incentives may also provide households “social protection” to maintain healthcare when income is uncertain. Compared to children and older adults, however, AYA face more numerous, diverse and intense barriers to ART adherence and retention, and have poorer treatment outcomes [5, 6]. The combination of incomplete development of neural pathways required for decision‐making, in particular those based on abstract goals, distal outcomes and cost–benefit calculations [7], and, contextual factors, such as peer relationships, financial dependency and school restrictions, constrain decision‐making during this critical developmental period. Financial incentives have demonstrated short‐term behavioural effects on AYA for HIV prevention, but to date, optimum strategies for delivering financial incentives to improve long‐term HIV treatment outcomes in this group are unclear [8, 9, 10, 11, 12].

Mixed methods research is being applied with increased frequency in implementation research to explore dimensions of acceptability, preference and choice [13]. Qualitative methods offer depth and richness of perspectives, while surveys attempt to quantify or categorize acceptability and satisfaction. Routine quantitative measures of acceptability, tend however, to provide superficial assessments [14], while economic preference elicitation methods offer techniques, deeply rooted in consumer behaviour theory, that demonstrate which features make an implementation strategy more or less acceptable, through the quantification of relative preferences for strategy features. Discrete choice experiments (DCEs), in particular, are gaining prominence as an implementation science tool—now applied extensively in HIV research—for developing strategies and for policy prioritization [15, 16, 17]. Additional qualitative research can be used both to inform DCE design and contextualize DCE findings—in combination such mixed methods generate a broad perspective of what is important to stakeholders, including how they make trade‐offs between service features. Given the unique challenges faced by AYA, understanding the dimensions of what would make an incentive delivery strategy most acceptable or desirable, as well as how this varies across the population, can contribute to the development of a patient‐centred implementation strategy.

To inform the design of financial incentives within a parent trial aimed at improving retention in care among AYA [18], we conducted a mixed methods study incorporating relative preferences from a discrete choice survey (DCE) with a qualitative focus group discussion (FGD) to explore preferences for incentive delivery.

## 2 METHODS

### 2.1 Study setting

This study was conducted at three health facilities (Lumumba Health center, Kisumu County Hospital and Ahero Sub County hospital) located in the city of Kisumu, Kenya (Kisumu County, Nyanza region) between 28 October and 12 November 2020. Kisumu County is among the highest HIV burden counties in Kenya with a prevalence of 16.3%, far higher than the national prevalence of 4.9% [19]. All sites had adolescent centres providing youth‐friendly services supported by the Family AIDS Care and Education Services (FACES). At the time of the study, study sites reported 30% of AYA (aged 14–24 years) as male and 70% as female, 38% were aged 14–17 years and 62% were 20–24 years of age. In 2020, viral suppression (VL <1000 copies/ml) among adolescents (15–19 years) in the FACES programme was 90%.

### 2.2 Study design and sample size estimation

The study comprised of a DCE survey and an FGD, both exploring preferences for financial incentive delivery among AYA living with HIV. The data for both the quantitative and qualitative components were collected simultaneously [20].

For the DCE, we selected potential attributes related to incentive delivery (based on literature review and discussion with experts in the field) and then prioritized the attributes, removed inappropriate attributes and refined wording to ensure attributes were culturally tailored [21, 22]. We selected five attributes including: incentive value (100 KSH [∼1 USD], 300 KSH [∼3 USD] or 500 KSH [∼5 USD]), frequency of incentive disbursement (at each clinic visit or annually as a cumulative payment), eligibility to receive the incentive (only those virally suppressed or all AYA), who can collect the incentive (only the AYA themselves and someone nominated by them) and the method of incentive distribution (cash, mobile money transfer, cellular airtime or shopping voucher) (Table 1). We generated a D‐efficient DCE design, ensuring level balance, balanced overlap and near orthogonality [23, 24, 25, 26]. Each respondent answered nine randomly ordered choice tasks and one fixed choice task (a dominant scenario to assess internal validity), across 200 randomly allocated choice experiment versions. We estimated the DCE sample size based on the formula *N* >500*c*/(*t* × *a*), where *c* represents the largest number of levels for any attributes, *t* represents the number of choice tasks and *a* represents the number of alternative scenarios: requiring a sample size of 125 participants ([500 × 4]/[8 × 2]) for mean preferences (with no interactions between attributes). We further conducted simulated data logit efficiency tests to ensure parameter estimate standard errors remained <0.05 for the main analysis. With these considerations and to allow for subgroup analysis, we increased the sample size to require 200 participants. Sawtooth Lighthouse Studio (version 9.11.0) was used for DCE design and sample size logit efficiency tests.

**Table 1:** Incentive features (attributes) presented in the DCE

Attribute	Attribute levels
The value of the gift	100 KSH (∼1 USD)
	300 KSH (∼3 USD)
	500 KSH (∼5 USD)
When you receive the gift	At each clinic visit
	At the end of each year (saved at each clinic visit)
Who is eligible for the gift	All youth attending the ART clinic
	Only youth who attend clinic visits on time and are virally suppressed
Who collects the gift	Only you
	You or a person you have elected
How the gift is distributed	Cash
	mPesa (mobile money payment)
	Airtime
	Shopping voucher

A semi‐structured guide was developed to moderate FGD discussions and included focused questions and prompts (File S1). Topics in the guide covered general financial challenges of AYA, with a specific focus on financial incentive preferences, including preferred value, mode of delivery, potential use, recipient of the incentive and potential adverse effects of the incentive.

### 2.3 Recruitment

Recruitment followed eligibility criteria for the parent trial: AYA living with HIV aged between 14 and 24 years, receiving HIV care in the selected clinic, with a disclosed HIV status and living in Kisumu for at least 6 months were approached (using consecutive sampling) during routine clinic visits to participate in the DCE. For the FGD, participants were purposefully selected by community health assistants to identify information‐rich AYA (information‐rich participants can yield deep insights on the basis of having information on what the researchers wish to understand rather than provide a generalizable understanding of the study topic) and referred to study staff for eligibility screening. FGD participants were also purposively recruited to ensure age and sex balance. AYA participating in the DCE and FGD were reimbursed $5 (average transport costs within the study region).

### 2.4 Data collection

The DCE survey was developed in English and translated into two local languages (Kiswahili and DhoLuo). Trained research assistants consented ‐ AYA for DCE participation. The DCE was interviewer administered on tablets, in‐person, at the health facility (using COVID‐19 precautions). The survey comprised 22 questions, 12 socio‐demographic questions and 10 DCE questions.

The FDG was conducted online via Zoom due to COVID restrictions. The recruited AYA who did not have access to an internet‐enabled smartphone were asked to come the study offices to use a laptop/tablet in a private room. Following the interview guide, two qualitative interviewers facilitated the FGD, one moderator and another note taker. The FGD was audio recorded, and notes were taken to supplement the recordings. The FGD was initiated with video and audio, allowing moderators and participants to identify one another. After introductions, cameras were turned off for the remainder of the session due to internet bandwidth concerns. The AYA opted to use a mix of English and Kiswahili to increase comfort in expression. An initial demonstration on the use of Zoom functions (i.e. muting, hand raising and video) was conducted, and for those who opted to come to the facility to use study laptops/tablets, IT staff were available to resolve technical issues. AYA who participated remotely were asked to ensure privacy and reduce interruption. The FGD took approximately one and a half hours.

### 2.5 Analysis

For the DCE survey main effects analysis, we conducted mixed logit regression models with dummy coded data [27]. The resulting mixed logit coefficients can be interpreted as the strength of the relative preference for the attribute comparison, with positive coefficients representing positive preferences (desirable) and negative coefficients representing negative preferences (undesirable), and standard deviations representing preference heterogeneity. We calculated the relative importance of attributes as the utility range for a specific attribute divided by the total summative utility range for all attributes (as a percentage). Interaction between demographic or treatment characteristics (age, sex, socio‐economic status, schooling status, home location and viral load suppression), and relative preferences were evaluated by multiplying each covariate with dummy coded attribute levels to generate interaction terms which were included in mixed logit regression models. Latent class analysis was conducted using conditional logit models and final model selection was based on model fit criterion (Akaike and Bayesian information criterion) for a two‐ and three‐class model, mean probability of group membership and qualitative exploration [28]. Willingness to pay analysis, for exploration of trade‐offs within latent class groups, was conducted after establishing linearity of attribute levels for incentive value using Lowess plots. Generalized linear models with a log‐link function were used to estimate risk ratios with 95% confidence intervals for predictors of latent class membership. We conducted a sensitivity analysis to determine if preferences were affected by response quality, poor response quality was based on selecting the alternative to the dominant scenario in the fixed choice task or straight lining (selecting the first scenario for each question). Stata version 16 was used for DCE analysis.

For FGDs, notes were expanded immediately after the discussion and stored with audio recordings. Audio files were subsequently transcribed and translated into English. Transcripts and notes constituted study data for analysis. Two qualitative researchers iteratively developed an initial coding framework that guided a further coding process. Transcripts were double coded, applying both a deductive approach, based on the potential incentive features of interest (value, frequency, recipient, format and eligibility), as well as an inductive approach as new codes emerged. Discrepancies in the application of the codes were resolved through discussion in a series of meetings. Further thematic memos were generated to record emergent themes. This approach was used to identify themes related to preferences for financial incentives, as well as potential challenges with incentive distribution.

A mixed methods convergence approach was used to triangulate results from the FGD and DCE [20]. The conclusions from the qualitative FGD were compared with DCE findings to identify how service delivery preferences compared across methods, to highlight both similarities as well as unique insights emerging from each technique.

### 2.6 Ethical considerations

The study was approved by the Kenya Medical Research Institute (KEMRI) and Washington University in St Louis institutional review boards. Participants provided written and verbal informed consent. For adolescents who were not accompanied, verbal consent was obtained from their caregivers through a phone call. Assent was obtained from participants who were below 18 years old, and their caregivers/parents provided written and verbal consent.

## 3 RESULTS

Of the 199 AYA who completed the survey, the median age was 18.4 years (interquartile range: 16.4–21.5 years), 133 (67%) were female sex, 101 (56%) were in primary or secondary school, 99 (50%) reported some food insecurity and 146 (73%) lived in an urban setting (Table 2).

**Table 2:** Participant characteristics (*N* = 199)

Participant characteristics		*n* (%)
Age	14–17 years	91 (46%)
	18–20 years	52 (26%)
	21–24 years	56 (28%)
Sex	Female	133 (67%)
	Male	66 (33%)
Schooling	Primary school	45 (23%)
	Secondary school	66 (33%)
	Not in school^a^	88 (44%)
How often not enough food in household, in the last 12 months?	Never	100 (50%)
	Sometimes	86 (43%)
	Always	13 (7%)
Number of rooms in your primary residence	1	87 (44%)
	2	57 (29%)
	3 or more	55 (28%)
Place where patient or caregiver lives	Rural	53 (27%)
	Urban	146 (73%)
Facility	Lumumba sub‐county hospital	83 (42%)
	Ahero county hospital	50 (25%)
	Kisumu county hospital	66 (33%)
Time on antiretroviral therapy (years; median and interquartile range)		7.86 (2.92–11.45)
Most recent viral load^b^	< 50 copies/ml	150 (75%)
	50 < 1000 copies/ml	28 (14%)
	> = 1000 copies/ml	14 (7%)
	Missing	7 (4%)

^a^ Includes those in college or university.

^b^ Viral load measurement conducted median 28 days prior to survey (IQR: 84 day – 0 days prior to survey).

### 3.1 Relative preferences

**Figure 1:**
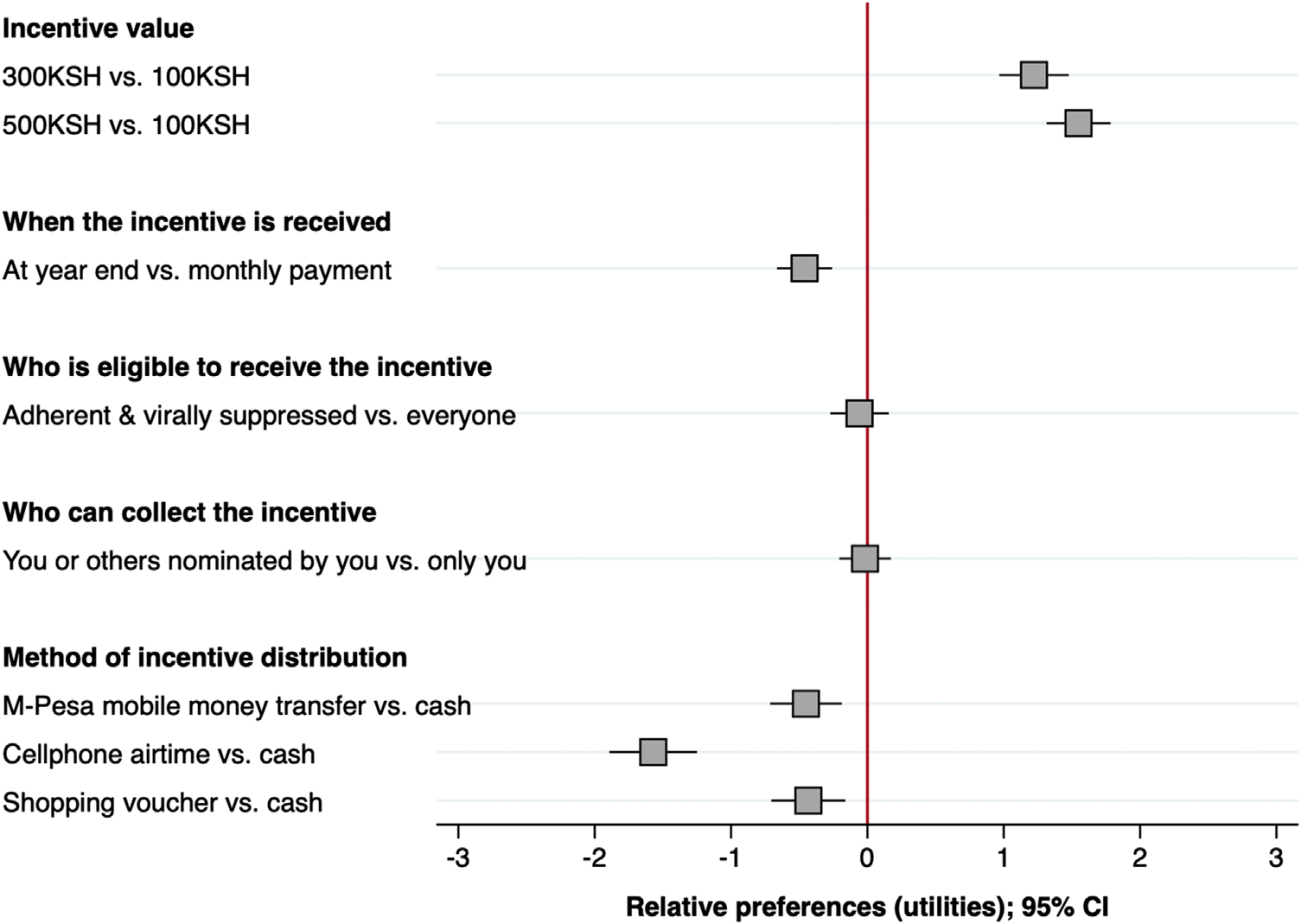
Relative preferences for incentive distribution strategies among all AYA (*N* = 199). Negative relative preferences represent what participants do not prefer; positive relative preferences represent what participants do prefer. The relative preference value represents the strength of preference relative to the baseline attribute level. Mixed logit model. CI, confidence interval.

Relative preferences across the population (Figure 1 and File S2) demonstrated that on average AYA preferred to receive a higher incentive amount than 100 KSH but that there was little difference between receiving 300 KSH (relative preference: 1.20; 95% CI: 0.96–1.45) or 500 KSH (relative preference: 1.52; 95% CI: 1.29–1.74). AYA did not want to receive incentives at the end of the year compared to monthly disbursements (relative preference: −0.51; 95% CI: −0.72 to −0.31). AYA found it equally acceptable that incentives were only provided to those who were suppressed and adherent, compared to everyone (relative preference: −0.07; 95% CI: −0.28 to 0.14), and that they themselves or someone they nominated collect the incentive on their behalf (relative preference: 0.00; 95% CI: −0.19 to 0.18). The preferred method of incentive distribution was cash, which was strongly preferred to cell phone airtime (relative preference: −1.52; 95% CI: −1.81 to −1.23) and moderately preferred to mobile money (relative preference: −0.42; 95% CI: −0.69 to −0.15) or shopping vouchers (relative preference: −0.40; 95% CI: −0.67 to −0.13). Sensitivity analyses showed no difference in preferences when restricted to high‐quality responses (*N* = 168) versus the total cohort (File S3). Calculation of relative attribute importance revealed that after incentive value (56%)—distribution method (32%) and frequency of distribution (10%) were the most important attributes, with eligibility (3%) and person collecting the incentive (3%) showing relatively low importance.

### 3.2 Interactions with AYA characteristics and viral load status

Analysis of interactions between relative preferences and participant demographic characteristics and viral load revealed that those who were older (18–24 years) preferred mobile money transfers to cash (relative preference: 0.63; 95% CI: 0.12–1.14) compared to those who were younger (14–17 years) who preferred cash (relative preference: −0.76; 95% CI −1.33 to −0.19) to mobile money. Those who were older also preferred monthly rather than yearly incentive disbursements (relative preference: −0.67; 95% CI: −1.09 to −0.25) compared to those who were 14–17 years, who preferred yearly disbursements (relative preference: 0.47; 95% CI: 0.05–0.90). This was mirrored in the analysis of interactions with schooling status which demonstrated that those not attending school preferred monthly disbursements (relative preference: −0.67; 95% CI: −1.12 to −0.22), while those still attending school preferred yearly payments (relative preference: 0.56; 95% CI 0.15–0.97). There was also evidence that females were more willing to accept shopping vouchers instead of cash (relative preference: 0.55, 95% CI: −0.05 to 1.14) compared to males who preferred cash (relative preference: −0.73, 95% CI: −1.41 to −0.06) to shopping vouchers. There was little correlation between the two measures of socio‐economic status—food availability and the number of rooms in the home (Pearson's correlation coefficient: −0.23)—we, therefore, evaluated interactions with food availability only. There was no interaction between this measure of food insecurity (sometimes or always did not have enough food) versus no food insecurity (always enough food) and AYA preferences. Evaluation of interactions with recent viral load status (viral load <50 copies/ml) also did not reveal any substantial difference in preferences for AYA.

### 3.3 Latent class analysis

**Figure 2:**
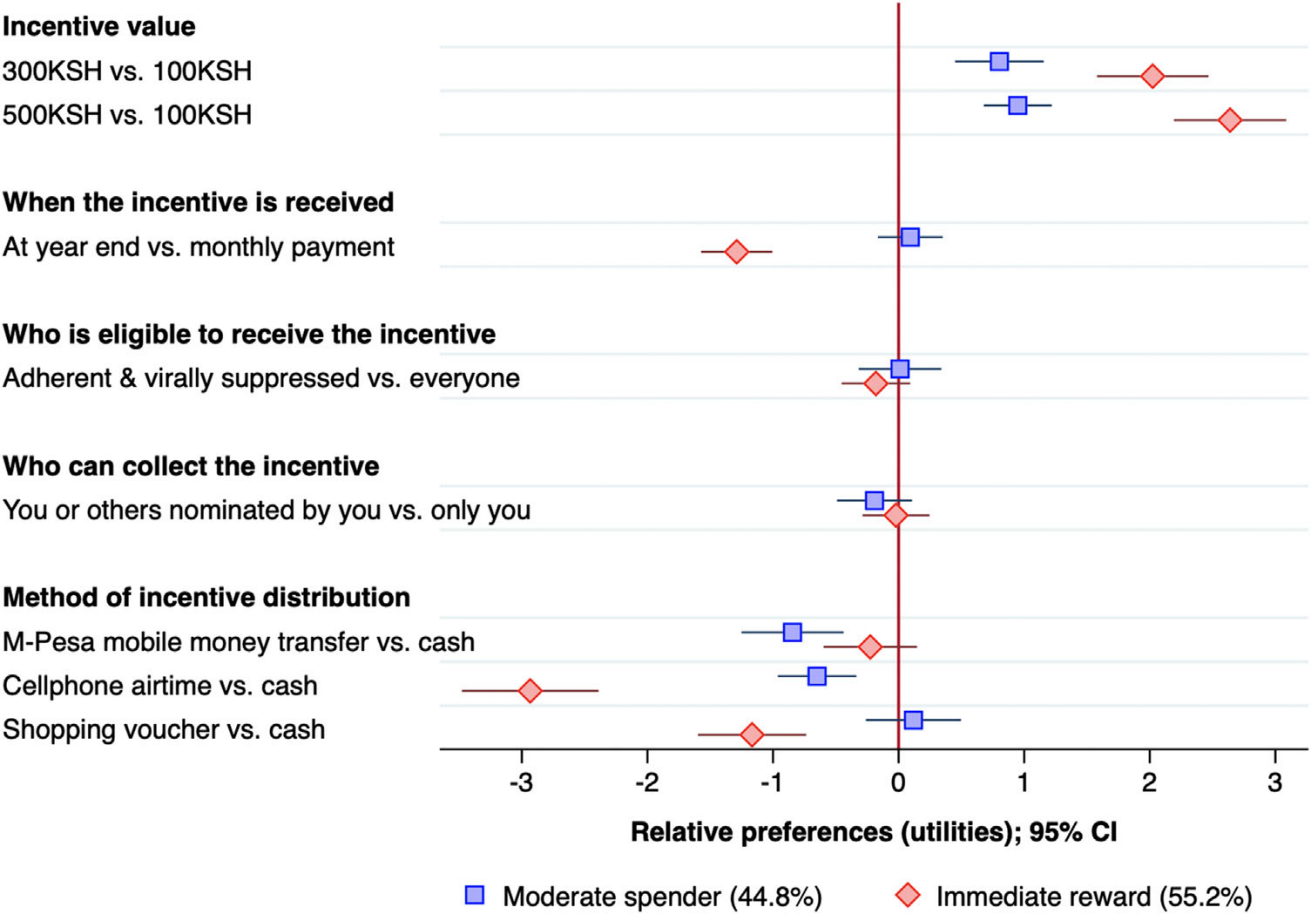
Relative preferences for incentive distribution strategies, by latent class preference group. Negative relative preferences represent what participants do not prefer; positive relative preferences represent what participants do prefer. The relative preference value represents the strength of preference relative to the baseline attribute level. Mixed logit model by latent class membership. CI, confidence interval.

We identified two latent class groups, revealing two distinctive preference phenotypes in the AYA population (Figure 2 and File S4a). One group (44.8%), who were focused on “immediate reward,” had strong preferences for the highest value incentives (KSH 500 vs. KSH 100: relative preference 2.66; 95% CI: 2.18–3.14), receiving money on a monthly basis (relative preference: −1.31; 95% CI: −1.63 to −0.99) and were equally willing to receive a cash incentive or mobile money transfer (relative preference: −0.22; 95% CI: −0.65 to 0.21), rather than cell phone airtime (relative preference: −2.92 95% CI: −3.49 to −2.34) or shopping vouchers (relative preference: −1.18; 95% CI: −1.61 to −0.74). In a willingness to trade analyses, this translated to a willingness to trade up to 204 KSH (95% CI: 159–250 KSH) of a 500 KSH incentive to receive incentives on a monthly rather than annually basis. The second group 55.2% of the population—the “moderate spender” group—were overall more accepting of lower value incentives and year‐end disbursements (relative preference: 0.12; 95% CI: −0.15 to 0.39). Moderate spenders equally preferred cash or shopping vouchers (relative preference: 0.12 95% CI −0.25 to 0.49) but demonstrated a preference for cash rather than mobile money transfers (relative preference: −0.78; 95% CI −1.17 to −0.40) or cell phone airtime (relative preference: −0.59; 95% CI: −0.91 to −0.27). In the multivariate model including age, sex, viral load and food security (File S4b), age was the strongest predictor of latent class group membership, with those who were older (18–24 years compared to 14–17 years) more likely to belong to the “immediate reward” group (RR 1.45; 95% CI: 1.08–1.95; *p* = 0.006).

### 3.4 Focus group discussion

A total of eight AYA took part in the FGD. Five were between the age of 14–17 years, three were 18–24 years of age and half (4) of the AYA were male. Table 3 presents the FGD findings.

**Table 3:** Related themes and quotes from focus group discussion

Theme	Summary	Relevant quotes
Incentive value	Preferences for incentive value ranged from 200 to 1000 per visit but for the majority, KSH 500 was acceptable	*The amount I see to be appropriate is let's say five hundred that can be appropriate…* R2 (M, 14–17 years) *My opinion is 500… this will motivate me to take my medication on time*. R7 (F, 18–24 years)
When the incentive is received	Participants in boarding school preferred accrued incentive disbursements	*I can suggest that it be given after a period of time like when the individual can be available like for those in boarding, they be given after a period of time when they will be available to avoid issues such like the parent received and they didn't reach the intended*. R6 (F, 18–24 years)
Who is eligible to receive the incentive	There was some support for providing incentives to only those who were adherent	*For those who are not adhering they have been promised if their viral load will be at zero they will be given a certain amount,…*. R2 (M, 14–17 years)
Who can collect the incentive	Participants preferred to receive funds themselves, in part due to mistrust of caregivers	*… so if it is given to the caregiver, they can lie to you that maybe they haven't received the money, they haven't sent the money and yet they had been sent for, so I propose it be sent to us because we know what we need*. R5: (M, 14–17 years) *…you give us the money and then we decide if we are going to give it to our caregivers a little bit or not* R4:
Method of incentive distribution	Distribution preferences varied considerably	*Let it be given in cash*. R2 (M, 14–17 years) *If transferred via their phones it will be at least unlike giving them the hard cash… because for some if they go home with the cash, they will be questioned where they got it from*. R8 (F, 14–17 years) *I would say something like shopping or a present, it is not a must it be cash like they could be taken to the supermarket and be told to pick anything they want of a certain amount, then they pay for or a gift, it is not a must cash*. R6 F (18–24 years)
Incentive use	The AYA envisioned several purposes for funds, including starting small businesses and purchasing luxury items	*The money can help them in many ways, one can open jobs, others it can help as transport, for others it can help them buy the things that they need, also fruits…*. R1 (Male (14–17 years) *…if I get myself 500 or 1000 bob, I will go get that kind of cloth that I want, that kind of shoes that I want, I want to get that kind of makeup that I want…*. R4: Female (18–24 years
Concerns around receiving incentive	Influx of funds could raise suspicions in their community	*…maybe you had no money and you've been given the 500 and maybe you owed someone and they are pressurizing you that they need it then, and you tell them you have no money, so he decides to attack you with his group…*. R2 (M, 14–17 years) *… in the village most of the people may investigate where you get money from… you know when they get to hear about the word clinic it means you are HIV positive, you will face stigma because of the small issues*. R1 M (14–17 years)

AYA preferred incentive values of KSH 500 or more, but lower values remained acceptable for some. Participants also suggested that for those who lived far from the clinic, higher value incentives may be considered for transport costs. Most AYA preferred receiving funds directly, citing concerns that the funds would not reach them if others were to collect on their behalf, though some in boarding schools were willing to have parents collect their incentive while away. Intermittent disbursements of accrued incentives were also most desirable to those in boarding school. Cash was the preferred incentive delivery format, but for AYA with mobile phones, electronic money transfers were preferred due to the convenience and privacy it afforded. AYAs had varied intentions for the use of incentive funds (for transport, to support household expenses or to purchase luxury items). AYA also raised a few concerns regarding how a sudden influx of funds may raise suspicions in the family or community.

## 4 DISCUSSION

Findings from this mixed methods study including 207 AYA living with HIV in Kisumu, Kenya revealed that on average AYA prefer to receive high‐frequency cash incentives compared to other distribution methods to support their retention in HIV care, but also that preferences vary—particularly by age and boarding school attendance. Latent class analyses identified two preference phenotypes, each comprising about half of the population—the older “immediate reward” group who wanted monthly access to high‐value incentives in the form of cash or mobile money transfers and the younger “moderate spenders” group who accepted lower value, simple incentive delivery systems (such as cash or shopping vouchers), distributed at any time point. AYA envisioned using the funds for a wide variety of activities but also raised a few concerns that changes in financial status could be viewed with suspicion in the community.

Developing age‐specific incentive delivery approaches could improve the effectiveness of incentive delivery strategies. Age was the strongest driver of preference heterogeneity, those who were 18–24 years of age were more likely to belong to the “immediate reward” latent class preference group who desired high‐value incentives in an unconstrained format (i.e. cash or mobile money transfer), but who were also willing to accept lower value incentives to ensure more frequent and immediate disbursements—invoking the behavioural economic principle of temporal discounting—less money now is more valuable to individuals than more money later [29]. This tendency to see future rewards as less valuable than more immediate rewards has previously been identified among people living with HIV (PLWH) and high‐risk groups in rural Ugandan for HIV testing uptake and in Mexico for pre‐exposure prophylaxis uptake [30, 31]. In contrast, the preferences of the “moderate spenders” (50%), who were on average younger (14–17 years), were more tempered. This group's generally more moderate preferences were related to school attendance, which reduced the ability to collect incentives and possibly to use funds—contextual factors that could modify the effectiveness of incentive disbursements in younger AYA.

The provision of cash incentives appeared to be the most equitable and versatile incentive disbursement method for AYA. Although mobile money transfers were preferred by some, the lack of phone access for others made this approach less universally acceptable. AYA reported a range of intended uses for incentive funds; to support household expenses, pay for transport or personal effects. Across all groups, shopping vouchers and cellular airtime were relatively undesirable. AYA living with HIV in South Africa similarly show preferences for cash incentives delivered in‐hand at clinic visits rather than electronically or through gift vouchers [32].

Conditional incentives can be effective in improving ART adherence if individuals can control their adherence. In cases where adherence is governed by structures beyond the individuals’ control—as is often the case for AYA—including family interference or the school environment, unconditional incentives may, however, be more equitable. Participants in our study had overall high adherence (89% with a viral load <1000 copies/ml), limiting the ability to conduct subgroup analysis by the level of viral suppression. A DCE conducted among AYA in South Africa with lower levels of retention and adherence demonstrated a strong preference for unconditional incentives relative to conditional incentives [32]. Both incentivization approaches have been shown to be effective in influencing behaviour change in adults, operating either as a nudge towards a health behaviour or through the provision of social protection to undertake specific health behaviours [2, 3]. For AYA living with HIV, a deeper context‐specific exploration of preference drivers and the locus of control for ART adherence could help determine which approach would be most effective [33].

Harms related to the provision of financial incentives to AYA are rarely reported in the literature [33, 34, 35]. AYA participating in the focus group discussion in our study raised concerns about how knowledge of a sudden influx of funds could raise suspicions in the community and potentially make AYA targets for investigation or disclosure of HIV status. These findings suggest that future explorations of harms associated with the distribution of financial incentives and strategies for ameliorating these among AYA may be warranted.

Although the use of financial incentives to encourage health behaviours in sub‐Saharan Africa remains rare outside of research environments, the widespread success of integrating conditional cash transfers into public programmes in South America, to alleviate poverty, increase school attendance, childhood vaccination rates and preventative health behaviours, demonstrates the feasibility of implementing such programmes in low and middle income country settings [36, 37]. Implementation considerations for incorporating financial incentives into HIV programmes—particularly to influence long‐term health behaviours such as medication adherence—will, however, require broader context‐specific investigations of effectiveness, feasibility and sustainability in the sub‐Saharan Africa context.

The study was limited by the small sample size of the qualitative component of the research and the inherently hypothetical nature of the DCE choice scenarios, which may not have fully reflected how AYA make choices in real life. In addition, it was not possible to compare characteristics of AYA who declined to participate as these data were not captured during recruitment; however, the sex and age distribution, as well a level of viral suppression of AYA included in the DCE, mirrored those reported in programme data for youth in ART services in Kisumu during the study period.

Our study findings were strengthened by the use of a mixed methods approach to understand the general as well as relative acceptability of financial incentive delivery strategies for AYA in Kenya. For example, although the qualitative FGD data revealed a preference to receive funds directly rather than via caregivers, when quantified in the DCE relative to other incentive delivery features (such as incentive value and frequency of disbursement), incentive collection by caregivers was relatively more acceptable—such trade‐offs reveal the critical components of the incentive strategy for AYA. These insights demonstrate how qualitative data and econometric data provide complementary information on the acceptability of implementation strategies, by exploring several dimensions of acceptability—what is acceptable, for whom and under what conditions? Mixed methods preference research is an essential tool for developing patient‐centred implementation strategies that consider the role of preference heterogeneity and offer a more personalized public health perspective on implementation [13, 38].

## 5 CONCLUSIONS

The provision of a KSH 500 cash incentive, as opposed to other options, such as mobile money, vouchers or airtime, was the most uniformly acceptable incentive delivery format among AYA in this setting. Given, the marked heterogeneity of preferences for other incentive delivery features (recipient, disbursement frequency and mobile money payments)—particularly by age group—providing options that align with circumstances and life stage would be the most patient‐centred strategy for AYA.

## COMPETING INTERESTS

The authors declare no competing interests.

## AUTHORS’ CONTRIBUTIONS

IEW, EA, FA, SI, JLK, JN, AS, EN, EAB, SO, EHG and LA contributed to DCE attribute development and refinement, revision and fielding of the survey tool. IEW conducted DCE analyses and data triangulation. BO and ZK contributed to qualitative methodology, data collection, analysis and data triangulation. All authors contributed to manuscript development and revisions.

## FUNDING

LA and EHG are supported by the NIH R01 NR018801. IEW is supported by the NIH KL2 TR002346. The funders had no role in the study design, data collection and analysis, decision to publish or preparation of the manuscript.

## Supporting information


**Additional File 1**: FGD guide for conditional cash transfers.

**Additional File 2**: Mean population preferences all respondents; mixed logit model (*N* = 199).

**Additional File 3**: Sensitivity analysis: mean population preferences for good quality responses; mixed logit model (*N* = 168).

**Additional File 4a & 4b**: LCA Model fit and Predictors of Latent Class Membership.

## Data Availability

Original data are available on request from the author.
