# Supplementary material for: Adolescent and young adult preferences for financial incentives to support adherence to antiretroviral therapy in Kenya: a mixed methods study
Source: J Int AIDS Soc. 2022 Sep 15;25(9):e25979. doi: 10.1002/jia2.25979 (PMC9478044; doi:10.1002/jia2.25979)
Supplement: Supplementary file 1 — Additional File 1: FGD guide for conditional cash transfers. [file JIA2-25-e25979-s001.docx]

**Additional File 1: FGD guide for conditional cash transfers**

1. What would you say is the experience of adolescent and young people enrolled in HIV care in this community?
2. What are some of the challenges adolescent and young people face receiving care in this community?
3. Many adolescent and young people are unable to return to clinic for their follow up visits and therefore become lost to programs, what do you think are some of the reasons?
4. Describe to me the financial situations of young people aged 14-19?
5. Do adolescents age 14-19 have money?
6. Where do they get money? Can they earn money?
7. Is it different for males and females? (chama, merry go round, loan apps)
8. Do they borrow money? Do they have debts?
9. Where do they borrow money?
10. How do they use money?
11. Describe to me the financial situations of young people aged 20-24
    1. Do young people 20-24 have money?
    2. Is it different for males and females? Can they earn money?
    3. Do they borrow money? Do they have debts?
    4. Where do they get money?
    5. How do they use money?
12. If adolescents and young people have money,
    1. How do they share the money?
    2. Who do they share with? Is this different for males and females?
    3. Do parents know when young people have money?
    4. Do spouses/partners/boyfriends/girlfriends know when you have money?
    5. Who makes decisions about how the money is spent?
13. What do you think about giving adolescents conditional cash transfer that motivates appointment attendance and viral suppression? (Probes: How will it help? Why do you think it will not help?)
14. What is the appropriate amount to encourage the behavior (Clinic attendance and viral suppression)?

- For each visit?
- For each viral load result (2 per year)?

b. Why do you think this is an appropriate amount?

1. What concerns do you have about giving adolescents and young people money to support good HIV health outcomes? *Probes- incentive to fail, misuse of money, coercion*

9. To whom do you think this money should be disbursed to (Caregiver or AYA)?

1. Why do you think the money should be disbursed to this person?
2. How do we manage CTCs for younger participants?
3. At what age should we disburse the CCT to caregivers and not AYAs (patient)?
4. If given to the caregivers, how do we ensure that the AYA patient benefits from it?

10. What would be the appropriate means to disburse this money (cash? m-pesa? Save it?) Why do you think so?

1. What do you think can be done if someone comes to collect drugs on the patient’s behalf when a CTC would have been disbursed to the patient?
2. What if the AYA does not have their own phone line or unable to receive the funds directly?
3. Is a savings plan distributed near the end or applied to education, etc. motivating and feasible? Why do you think so?
4. To what extend do you think the money disbursed will be used to support the adolescent and young people to remain in care?
5. How will this money be used to help the adolescent and young people to remain in care?
6. What activities do you think will be supported with this money?
7. What challenges to you anticipate with receiving this money?
8. What are your thoughts about how this money can be better used to support adolescent and young people to remain in care?
