# Supplementary material for: Adolescent and young adult preferences for financial incentives to support adherence to antiretroviral therapy in Kenya: a mixed methods study
Source: J Int AIDS Soc. 2022 Sep 15;25(9):e25979. doi: 10.1002/jia2.25979 (PMC9478044; doi:10.1002/jia2.25979)
Supplement: Supplementary file 2 — Additional File 2: Mean population preferences all respondents; mixed logit model (N = 199). [file JIA2-25-e25979-s003.docx]

| Attribute | Mean Preferences | | | | Standard Deviation | | | |
| --- | --- | --- | --- | --- | --- | --- | --- | --- |
|  | Estimate | p-value | Low CI | High CI | Estimate | p-value | Low CI | High CI |
| Incentive 300 KSH vs. 100 KSH | 1.22 | <0.001 | 0.97 | 1.48 | 0.30 | 0.42 | -0.44 | 1.04 |
| Incentive 500 KSH vs. 100 KSH | 1.55 | <0.001 | 1.31 | 1.78 | -0.45 | 0.03 | -0.86 | -0.04 |
| Received year end vs monthly | -0.46 | <0.001 | -0.66 | -0.26 | 0.86 | <0.001 | 0.55 | 1.17 |
| Only adherent and virally suppressed vs everyone | -0.06 | 0.60 | -0.27 | 0.16 | 1.04 | <0.001 | 0.71 | 1.37 |
| Only youth receive versus other nominated person | -0.02 | 0.86 | -0.21 | 0.17 | 0.69 | <0.001 | 0.42 | 0.96 |
| Mpesa vs cash | -0.45 | <0.001 | -0.71 | -0.19 | 0.69 | <0.001 | 0.23 | 1.15 |
| Airtime vs cash | -1.57 | <0.001 | -1.89 | -1.25 | -0.45 | 0.20 | -1.13 | 0.23 |
| Voucher vs cash | -0.43 | <0.001 | -0.70 | -0.16 | 0.78 | <0.001 | 0.35 | 1.20 |

**Additional file 2: Mean population preferences all respondents; mixed logit model (N =199)**
