# Supplementary material for: Adolescent and young adult preferences for financial incentives to support adherence to antiretroviral therapy in Kenya: a mixed methods study
Source: J Int AIDS Soc. 2022 Sep 15;25(9):e25979. doi: 10.1002/jia2.25979 (PMC9478044; doi:10.1002/jia2.25979)
Supplement: Supplementary file 3 — Additional File 3: Sensitivity analysis: mean population preferences for good quality responses; mixed logit model (N = 168). [file JIA2-25-e25979-s004.docx]

**Additional file 3: Sensitivity analysis: mean population preferences for good quality responses; mixed logit model (N =168)**

| Attribute | Relative utilities | | | | Standard Deviation | | | |
| --- | --- | --- | --- | --- | --- | --- | --- | --- |
|  | Estimate | p-value | Low CI | High CI | Estimate | p-value | Low CI | High CI |
| Incentive 300 KSH vs. 100 KSH | 1.53 | 0.00 | 1.19 | 1.86 | -0.49 | 0.20 | -1.25 | 0.26 |
| Incentive 500 KSH vs. 100 KSH | 1.93 | 0.00 | 1.66 | 2.20 | -0.20 | 0.47 | -0.72 | 0.33 |
| Received year end vs monthly | -0.64 | 0.00 | -0.88 | -0.40 | 0.87 | 0.00 | 0.53 | 1.20 |
| Only adherent and virally suppressed vs everyone | -0.02 | 0.89 | -0.27 | 0.23 | 1.06 | 0.00 | 0.70 | 1.41 |
| Only youth receive versus other nominated person | -0.06 | 0.57 | -0.25 | 0.14 | 0.54 | 0.00 | 0.21 | 0.88 |
| Mpesa vs cash | -0.46 | 0.00 | -0.78 | -0.15 | -0.94 | 0.00 | -1.35 | -0.53 |
| Airtime vs cash | -1.69 | 0.00 | -2.04 | -1.35 | 0.35 | 0.29 | -0.29 | 0.99 |
| Voucher vs cash | -0.47 | 0.01 | -0.80 | -0.13 | 0.77 | 0.01 | 0.17 | 1.37 |
