# Supplementary material for: Adolescent and young adult preferences for financial incentives to support adherence to antiretroviral therapy in Kenya: a mixed methods study
Source: J Int AIDS Soc. 2022 Sep 15;25(9):e25979. doi: 10.1002/jia2.25979 (PMC9478044; doi:10.1002/jia2.25979)
Supplement: Supplementary file 4 — Additional File 4a & 4b: LCA Model fit and Predictors of Latent Class Membership. [file JIA2-25-e25979-s002.docx]

**Additional file 4a & 4b: LCA Model fit and Predictors of Latent Class Membership**

Table 4a: LCA model fit statistics

| **Attribute** | **Utility estimates & Model fit – conditional logit latent class model** | | | | |
| --- | --- | --- | --- | --- | --- |
|  | **Two class model** | | **Three class model** | | |
|  | **Class 1**  **(44.8%)** | **Class 2**  **(55.2%)** | **Class 1**  **(25.1%)** | **Class 2**  **(45.3%)** | **Class 3**  **(29.7%)** |
| Incentive 300 KSH vs. 100 KSH | 1.45 (0.97 to 1.94) | 0.69 (0.43 to 0.96) | 1.45 (-0.75 to 2.14) | 1.36 (0.88 to 1.83) | 0.33 (-0.11 to 0.77) |
| Incentive 500 KSH vs. 100 KSH | 1.96 (1.42 to 2.50) | 0.82 (0.55 to 1.09) | 1.47 (0.85 to 2.09) | 1.9 (1.40 to 2.40) | 0.59 (0.21 to 0.98) |
| Received year end vs monthly | -0.95 (-1.29 to-0.60) | 0.04 (-0.22 to 0.30) | 0.09 (-0.38 to 0.53) | -1.02 (1.41 to -0.63) | 0.05 (0.31 to 0.42) |
| Only adherent and virally suppressed vs. everyone | -0.14 (-0.48 to 0.20) | 0.06 (-0.19 to 0.32) | 0.69 (0.05 to 1.33) | -0.23 (-0.55 to 0.98) | -0.38 (-0.72 to -0.03) |
| Only youth receive vs. other nominated person | -0.05 (-0.36 to 0.27) | -0.07 (-0.19 to 0.32) | 0.58 (0.19 to 0.97) | -0.18 (-0.55 to 0.35) | -0.43 (-0.82 to -0.30) |
| Mpesa vs. cash | -0.14 (-0.57 to 0.29) | -0.58 (-0.92 to -0.24) | -0.41 (-1.00 to 0.18) | -0.10 (-0.55 to 0.36) | -0.81(-1.37 to -0.25) |
| Airtime vs. cash | -2.19 (-2.85 to -1.54) | -0.58 (-1.01 to -0.28) | -1.17 (-1.94 to -0.40) | -2.11 (-2.76 to -1.46) | -0.56 (-0.99 to -1.27) |
| Voucher vs. cash | -0.86 (-1.31 to -0.40) | 0.02 (-0.34 to 0.38) | -0.96 (-1.57 to -0.36) | -0.66 (-1.13 to -0.19) | 0.46 (0.01 to 0.91) |
| **Model fit statistics** |  | |  | | |
| Log likelihood | -1123.35 | | -1103.73 | | |
| AIC | 2280.70 | | 2256.45 | | |
| BIC | 2334.89 | | 2342.33 | | |
| CAIC | 2351.89 | | 2368.33 | | |
| Mean probability of class membership | 84.42% (sd: 14.29%; range: 51.32% to 100%) | | 79.89% (sd: 17.03%; range: 41.46% to 100%) | | |

AIC: Akaike information criterion; BIC: Bayesian information criterion; CAIC: Conditional Akaike information criterion

4b: Predictors of Latent Class Membership

| Predictor | | RR | Low CI | High CI | p-value |
| --- | --- | --- | --- | --- | --- |
| Age | 14-17 years | 1.00 | 1.08 | 1.93 | 0.006 |
|  | 18-24 years | 1.45 |  |  |  |
| Sex | Female | 1.00 | 0.82 | 1.42 | 0.601 |
|  | Male | 1.08 |  |  |  |
| Food security | Always food | 1.00 | 0.75 | 1.26 | 0.833 |
|  | Sometimes/Always not enough food | 0.97 |  |  |  |
| Viral load | VL < 50 copies/ml | 1.00 | 0.94 | 1.63 | 0.144 |
|  | VL >= 50 copies/ml | 1.23 |  |  |  |

*VL – viral load
